# Response to the letter regarding Agreement and systematic bias between QuantiFERON chemiluminescent immunoassay and QuantiFERON enzyme-linked immunosorbent assay in the detection of latent tuberculosis infection: A systematic review and meta-analysis

**DOI:** 10.1016/j.ijregi.2026.100855

**Published:** 2026-02-11

**Authors:** Bwambale Jonani, Felix Bongomin

**Affiliations:** 1 Department of Immunology and Molecular Biology, School of Biomedical Sciences, College of Health Sciences, Makerere University, Kampala, Uganda; 2 Department of Clinical Laboratory, Sebbi Hospital, Wakiso District, Uganda; 3 Department of Medical Microbiology and Immunology, Faculty of Medicine, Gulu University, Gulu, Uganda

**Keywords:** QuantiFERON chemiluminescent immunoassay, QuantiFERON enzyme-linked immunosorbent assay

Dear Editor,

We thank Li, Zhang, and Shang for their thoughtful commentary [5], on our recent systematic review and meta-analysis [1], in which we evaluated the agreement and systemic bias between LIAISON QuantiFERON-TB Gold Plus (herein referred to as QFT-CLIA) and QIAGEN’s QuantiFERON-TB Gold Plus enzyme-linked immunosorbent assay (ELISA) (herein referred to as QFT-ELISA) in detecting latent tuberculosis infection [1]. Their observations regarding borderline results, the heterogeneity across populations, and the “very low” certainty of evidence are point on [5]. We welcome the opportunity to clarify and extend our findings with additional data.

First, we agree and maintain that analytical concordance does not automatically translate into clinical interchangeability. Their commentary rightly emphasizes the importance of discordant results near the diagnostic threshold of 0.35 IU/ml, something we had observed in our meta-analysis. We reported a mean discordance rate of 6.5%, with directional bias varying across studies. We concur that these discrepancies are clinically relevant, particularly, in low-incidence settings where false positivity may trigger unnecessary evaluations that are costly to the patient and the health care systems. Our recommendations for laboratories to conduct local verification studies was precisely intended to mitigate this risk.

The heterogeneity in agreement across risk populations is indeed a critical consideration. Our subgroup analyses demonstrated a lower concordance in high-risk groups (83%) than in the low-risk groups, where the concordance was 91%. We agree that interpretive algorithms must integrate pre-test probability rather than rely on uniform thresholds. This is especially pertinent in immunocompromised populations, where discordance may be amplified.

We further acknowledge the limitations of the primary studies included in our meta-analysis. The “very low” GRADE certainty reflected incomplete reporting, lack of blinding, and non-randomized designs. Although our synthesis provided a valuable overview, it also highlighted the urgent need for prospective, longitudinal studies linking platform discordance to patient outcomes.

Several authors have already proposed strategies to manage borderline or discordant QuantiFERON results. Buron and Banaei [2] demonstrated that the QFT-CLIA platform tends to yield inflated interferon-gamma (IFN-γ) values in the borderline range and recommend a repeat-testing algorithm or confirmatory ELISA in low-incidence settings to prevent against false positives. They demonstrated this using 77 false-positive samples originally tested with QFT-CLIA. When they retested QFT-CLIA false positives (TB1 or TB2 range, 0 to ≤1.3 IU/mL) with ELISA, the false positivity reduced by 84.3% (59 of 70). This was in contrast to when they retested with CLIA, which only resulted in reduction of false positivity rate by 10.4% (8 of 77) [2]. Similarly, Ruiz-Tagle et al*.* [3] showed that discordance clusters around the diagnostic cutoff in household tuberculosis (TB) contacts, emphasizing that interpretation must be contextual and incorporate pre-test probability. Of the 24 (16.7%) discordant pairs they had, all the 15 (62.5%) QFT-CLIA–positive/QFT-ELISA–negative samples had QFT-CLIA IFN-γ levels within borderline values (0.35-0.99 IU/ml). Only 9 (37.5%) had levels >0.99 IU/ml [3].

To complement these insights, we wish to share results from another evaluation we have conducted since the review. In a cohort of 261 pregnant women in Uganda, plasma samples initially tested with QFT-ELISA in 2021 and cryopreserved, were retested in 2025 using QFT-CLIA and QFT-ELISA [4]. Among the 231 viable samples in 2025, the overall agreement between the two platforms was 90.5% (Cohen kappa = 0.82). The Bland–Altman analysis revealed minimal systematic bias, and Deming regression confirmed linearity. Importantly, when we restricted the analysis to samples that remained stable (maintained their initial results as in 2021) across the 4 years of storage, agreement improved to 98.4% (Cohen kappa = 0.97), with 100% agreement for intermediate results [4]. These findings showed us that under controlled conditions, the two platforms are highly concordant and that storage affects disproportionately positive samples.

This additional data set reinforced our earlier conclusion in the meta-analysis that QFT-CLIA is a viable alternative to QFT-ELISA, particularly, for high-throughput antenatal care settings. At the same time, it underscores the commentary’s caution that “borderline results and population-specific factors require context-aware interpretation.” We agree that future research must determine whether discordant results predict differential risk of progression to active TB.

We appreciate the constructive critique and believe our new data provide further reassurance regarding these two platforms comparability while also affirming the need for careful implementation strategies. Together, these complementary perspectives advance the dialogue on optimizing latent TB infection detection in diverse epidemiologic contexts.

**Ethical approval:** Not applicable.

**Author contributions:** BJ: writing – original draft, validation, approval. FB: writing – review and editing, validation, approval.

**Consent to participate:** Not applicable.

**Consent for publication:** Not applicable.

**Declarations of competing interest:** The authors have no competing interests to declare.
